# Acute mastoiditis: A one year study in the pediatric hospital of Cairo university

**DOI:** 10.1186/1472-6815-10-1

**Published:** 2010-01-04

**Authors:** Mosaad Abdel-Aziz, Hassan El-Hoshy

**Affiliations:** 1 Department of Otorhinolaryngology, Faculty of Medicine, Cairo University, Cairo, Egypt

## Abstract

**Background:**

Acute mastoiditis is a serious complication of acute otitis media especially in the pediatric age group. This study reports the authors' experience in the treatment of children admitted with acute mastoiditis to the Pediatric Hospital of Cairo University throughout the year 2007, also we aimed to evaluate our current management of this serious disease.

**Methods:**

Nineteen children were included in this study, 11 females and 8 males, their ages ranged from 9 months to 11 years. All children were treated with intravenous antibiotic on initial admission, myringotomy was considered for cases that did not respond to medical treatment for 48 hours, while cortical mastoidectomy (with myringotomy) was reserved for cases that presented initially with subperiosteal abscess with or without post-auricular fistula, cases with intracranial complications and for cases that showed no response to myringotomy (after 48 hours). Follow up of the patients was carried out for at least 1 year.

**Results:**

Medical management alone was enough in 5 cases (26%); all of them had erythematous tender mastoid on first presentation. Seven cases (37%) needed myringotomy; 2 of them showed no response and they needed cortical mastoidectomy and the other 5 cases responded well except for 1 case that developed post-auricular subperiosteal abscess 2 months later necessitating cortical mastoidectomy with no evidence of recurrence till the end of the follow-up period. Seven cases (37%) presented with subperiosteal abscess and they needed cortical mastoidectomy with myringotomy; they showed no recurrence till the end of the study.

**Conclusion:**

Conservative management is an effective method in the treatment of non-complicated acute mastoiditis, but myringotomy should be considered if there is no response within 48 hours. Cortical mastoidectomy should be used in conjunction with the medical management in the treatment of complicated cases.

## Background

Acute mastoiditis is a serious complication of acute otitis media (AOM). It is more common in the pediatric age group as most patients are younger than 4 years [1], this higher incidence in younger age group reflects the peak age for AOM [2], however its incidence has been decreased since the revolution of antibiotic therapy [3]. Some recent literature indicated an increase of the disease incidence in last years especially in countries with less antibiotic prescription [2], while others reported that no increased incidence despite the national restriction guidelines of antibiotics prescription [4]. The disease my cause significant and even life-threatening complications beyond the tympanomastoid system; including subperiosteal abscess, Bezold's abscess, facial paralysis, suppurative labyrinthitis, meningitis, epidural and subdural abscess, brain abscess, lateral sinus thrombophlebitis, and otitic hydrocephalus [5].

The treatment of acute mastoiditis is variable, ranging from conservative management in the form of parenteral antibiotic therapy to myringotomy (with or without ventilation tube placement) to a more aggressive intervention in the form of mastoidectomy [3,6,7].

This study reports the authors' experience in the treatment of children admitted with acute mastoiditis to the Pediatric Hospital of Cairo University throughout the year 2007, also we aimed to evaluate our current management of this serious disease in the pediatric population.

## Methods

This study included all pediatric patients presented with acute mastoiditis throughout the year 2007 to the Otolaryngology Unit of the Pediatric Hospital of Cairo University. The subjects consisted of 19 children, 11 females and 8 males, their ages ranged from 9 months to 11 years with a mean age of 4 years and 7 months. Monthly distribution of the cases was noted and analyzed.

The following protocol was applied for all cases:

- History taking and Otolaryngological examination: the criteria for diagnosis of acute mastoiditis were post-auricular inflammatory signs, antero-inferior displacement of the auricle and evidence of an acute or recent episode of otitis media [8]. The history of previous attacks of AOM was recorded. Patients with incomplete data or in which the diagnosis was not conclusive were excluded. Also, patients suspected to have cholesteatoma were excluded.

- Microbiological study: culture was done for cases that presented with ear discharge before starting the antibiotic therapy, while it was not done for cases on antibiotic therapy as the result may be not reliable (i.e. antibiotics may affect bacterial growth). Also, cultures was not done for cases presented with intact tympanic membrane and even after myringtomy as all cases that underwent this maneuver were already on antibiotics.

- CT temporal bone was done for all cases; for detection of complications that may be present with acute mastoiditis, however MRI is more diagnostic for intracranial complications but the protocol of our institution is to do CT with contrast due to limited equipments.

- Management: on admission, all children were treated with intravenous antibiotics (Ceftriaxone in a dosage of 20-50 mg/Kg bodyweight for at least one week, but it can be changed after culture results). Myringotomy was considered for cases that did not respond to medical treatment for 48 hours, while cortical mastoidectomy (with myringotomy) was reserved for cases that presented initially with subperiosteal abscess -seen clinically or radiologically- with or without post-auricular fistula, cases with intracranial complications and for cases that showed no response to myringotomy alone (after 48 hours). Myringotomy was done without insertion of ventilation tube according to the protocol of our institute in cases of infection. Failure of treatment was defined by persistence of post-auricular pain and/or fever.

- Follow up of the patients was carried out for at least 1 year after discharge from hospital.

## Results

Among 10654 patients attending the Outpatient Clinic of the Pediatric Hospital of Cairo University in the year 2007; 19 children were admitted with acute mastoiditis, 11 females and 8 males, their ages ranged between 9 months and 11 years with a mean age of 4 years and 7 months. The parents of 12 children gave history of previous attacks of AOM, 1 child had history of post-aural incision for drainage of post-auricular abscess, and none of our patients had congenital syndromes, craniofacial anomalies or immunodeficiency. Only 7 children received oral antibiotics prior to presentation.

Monthly distribution of cases showed the highest peak of the disease in March (5 cases = 26%) followed by another peak in December (4 cases = 21%).

**Figure 1:**
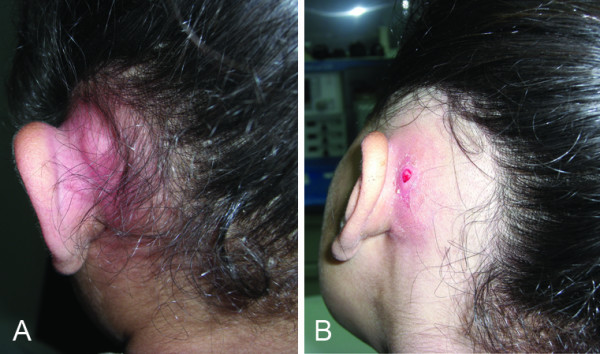
**Post-auricular abscess**. (A) without fistula and (B) with fistula.

Regarding the clinical presentation (Table 1); eight cases presented with erythema and tenderness over the mastoid with congested tympanic membrane in 6 of them, while the other 2 cases showed perforation and discharge. Seven cases presented with non- fluctuant post-auricular swelling with congested tympanic membrane in 1 case and perforation with discharge in 6 cases. Two cases presented with fluctuant post-auricular swelling (Fig 1) accompanied with tympanic membrane perforation and discharge. Two cases presented with post-auricular discharging fistula (Fig 1), all of them had perforated tympanic membrane with discharge.

**Table 1:** clinical features at presentation

Clinical feature	Number n = 19	Percentage
Post-aural erythema	8	42
Non-fluctuant post-aural swelling	7	37
Fluctuant post-aural swelling	2	10.5
Post-aural fistula	2	10.5
Congested tympanic membrane	7	37
Perforation and discharge	12	63

Bacterial culture of ear discharge was done for 12 cases; Streptococcus pneumoniae was identified in 5 cases and Streptococcus pyogenes in 3 cases, while Staphylococcus aureus was isolated in 1 case. No growth was obtained in 3 cases. Isolated organisms showed sensitivity to Ceftriaxone.

**Figure 2:**
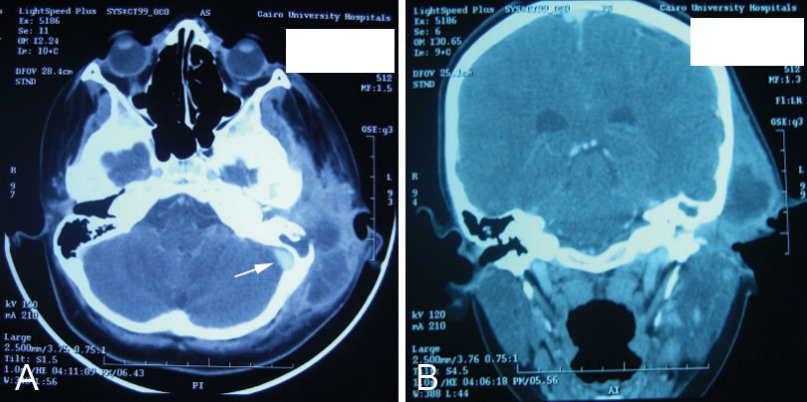
**CT of the skull shows subperiosteal abscess, (A) axial view and (B) coronal view with the arrow points to sigmoid sinus thrombosis**.

Radiological findings (CT) of mastoid bone showed subperiosteal abscess with destruction of cortex in 7 cases; one of them showed sigmoid sinus thrombosis in spite of absence of clinical manifestations (Fig 2).

Regarding the treatment outcome (Table 2); medical management alone was enough in 5 cases (26%); all of them had erythematous tender mastoid on first presentation. Myringotomy was done for 7 cases (37%); 2 of them showed no improvement and they needed cortical mastoidectomy, while improvement was achieved in 5 cases with no recurrence except in 1 case that developed post-auricular subperiosteal abscess 2 months later necessitating cortical mastoidectomy with myringotomy. Cortical mastoidectomy with myringotomy was done initially in conjunction with antibiotic therapy for 7 cases (37%) that presented with subperiosteal abscess with or without fistula (drainage of sigmoid sins thrombosis in 1 of them was carried out). All Cases that were subjected to cortical mastoidectomy -either initially or after failure of myringotomy- showed no recurrence till the end of the study.

**Table 2:** treatment outcome

Treatment	Number n = 19	Improved patients	Recurrence
Antibiotic therapy alone	5	5	0
Myringotomy	7	5	1
Cortical mastoidectomy	10	10	0

## Discussion

There is no doubt that the incidence of acute mastoiditis has decreased in the post-antibiotic era [7]. In 1946, House [9] pointed to an 80% decrease in the number of mastoidectomies performed after the introduction of sulfonamide. In 1959, Palva and Pukkinen [10] reported that 0.4% of AOM developed acute mastoiditis while in 1985 [11] the incidence was decreased to 0.004%. Recently, some authors observed that the number of children admitted to some hospitals with acute mastoiditis has risen [12-14]. However, Luntz et al [15] reported that the use of antibiotics is not a safe-guard against acute mastoiditis and it may lead to a latent (masked) mastoiditis. Also, Kvaerner et al [4] -In their registry based study on 399 Norwegian children- proved that the incidence of acute mastoiditis has not been increased in Norway despite the national restricted use of antibiotics in primary care.

In this study, 19 cases were diagnosed to have acute mastoiditis as a complication for AOM throughout the year 2007 in the Pediatric Otolaryngology unit of our institution; about 47% of cases were recorded in March and December. This may be explained by a high incidence of common cold and AOM in this period of the year [16].

Benito and Gorricho [17] reported that it is unknown whether failure to diagnose AOM promptly predisposes to acute mastoiditis, or middle ear disease in these cases takes a subclinical course; especially in young infants. On the other side Glynn et al [18] studied 29 children with acute mastoiditis; 69% of their patients had no previous history of AOM prior to presentation. However all our cases had middle ear pathology in the form of either congested or perforated tympanic membrane, which nearly matches with the results of Luntz et al [15] who reported pathological changes of the tympanic membrane in all of their cases except in 3.1%, also Bahadori et al [19] reported tympanic membrane changes in 95% of their cases.

The mastoid air cells are in continuity with the middle-ear space through the aditus ad antrum which is liable to be blocked by inflammatory reaction of the lining mucosa in AOM, resulting in an infected non-draining mastoid cavity while the middle ear can drain via the Eustachian tube. Thus the classical presentation of acute mastoiditis is a post-aural swelling with inflammatory signs [20].

In classic acute mastoiditis the protrusion of the auricle and retroauricular cellulitis is essential for diagnosis. At this stage or later the weakened tympanic membrane may rupture, and acute otorrhea can develop. Before destruction of mastoid bone (osteitis) there is a diffuse phlegmon (acute inflammation of the underlying connective tissue of the mastoid cavity).

Further progression of the inflammatory process within the mastoid space leads to destruction of the delicate trabecular system (coalescent mastoiditis). A subperiosteal abscess, pointing superior to or directly over the mastoid tip, occurs when the suppuration escapes through the thinned out mastoid cortex (or by thrombophlebitis of an emissary vein) and is entrapped by the periosteum and skin over the temporal bone and mastoid tip [19,21].

Regarding the clinical presentation of our patients; 42% showed post-auricular erythema, post-auricular swelling was detected in 58% (which was fluctuant in 10.5%, non-fluctuant in 37% and associated with fistula in 10.5%), while aural discharge with tympanic membrane perforation was detected in 63% of cases. Tarantino et al [3] detected post-auricular swelling in 100% of their cases with fluctuation in 25% of them; also the tympanic membrane was abnormal in all cases with perforation and discharge in 15%. De et al [20] reported that 85.7% of their cases had post-auricular swelling and 57.1% had aural discharge. However, the difference in the literature may be due to the stage of patients' presentation and the treatment taken prior to their presentation.

CT scan for our patients showed subperiosteal abscess in 37% and sigmoid sinus thrombosis in 5%. Bahadori et al [19] reported subperiosteal abscess in 13.5% of their cases, while Benito and Gorricho [17] studied 215 cases of mastoiditis and they reported complications -one or more- in 21 patients (9.76%); 15 patients had subperiosteal abscess, 1 had facial nerve palsy, and 9 had some sort of intracranial complication. However, radiological complications of mastoiditis are difficult to be estimated, so many authors do not recommend CT except for cases suspected to have intracranial complications and neurological manifestations [6,18,19].

Bacterial cultures were done for 12 of our patients that presented with aural discharge, no samples were taken for culture during myringotomy or mastoidectomy as the patients subjected to such procedures were already on antibiotics (Ceftriaxone) which may alter the results. Organisms were grown in 9 cases, Streptococcus pneumoniae was the most common bacterium identified followed by Streptococcus pyogenes, a finding that has been reported before by many authors [2,8,15,19]. Although Haemophilus influenzae is one of the most frequent pathogen in AOM in infants [2,22], it was not identified in culture of acute mastoiditis, however, Dudkiewicz et al [23], Dhooge et al [24] and Benito and Gorricho [17] reported that Haemophilus influenzae usually causes disease of the soft tissues and rarely progress to bone invasion. We selected Ceftriaxone as the antibiotic of choice as it is usually active against most of the causative agents responsible for acute mastoiditis that were mentioned before by many authors [2,8,15,19].

In our study; antibiotic therapy alone was enough in 5 cases (26%), while myringotomy was needed in 7 cases (37%); 2 of them showed no improvement and they needed cortical mastoidectomy. Cortical mastoidectomy was done initially in conjunction with antibiotic therapy for 7 cases (37%). All cases responded well for the protocol of treatment except 1 case (5.3%) that developed recurrence with subperiosteal abscess 2 months later and she was subjected to cortical mastoidectomy with no recurrence till the end of the study period.

A similar protocol of management was used by many authors; Tarantino et al [3] observed that medical treatment alone was enough in 65% of their cases, myringotomy was needed in 20% while 15% of cases needed mastoidectomy. They achieved 100% cure rate with this method of treatment. Papournas et al [25] reported that 23.2% of acute mastoiditis cases are usually in need for mastoidectomy. Katz et al [26] studied 116 cases; they noted that 12% of cases needed myringotomy while 28% needed cortical mastoidectomy.

Harely et al [7] studied 58 cases of acute mastoiditis, 29% of their children received antibiotic therapy as a sole treatment; this was effective in controlling disease (100% cure rate). Seventy one per cent needed adjunctive therapy in the form of myringotomy for 28 cases and mastoidectomy for 13 cases; the cure rate was 82% in the former and 92% in the latter, however, some of their children had cholesteatoma.

Holt and Gates [27] reported that the use of broad-spectrum antibiotics may lead to suppression of the presenting symptoms and signs of mastoiditis, causing the clinician to have a false sense of security following apparent resolution of the middle ear infection. The course may be so insidious that the first awareness of the mastoiditis may be following presentation of an intracranial complication such as meningitis, lateral sinus thrombosis, or brain abscess. Also, Luntz et al [15] concluded that masked mastoiditis may be caused by the use of antibiotics, so they recommended early myringotomy. However, strict follow-up of treated patients may help in the diagnosis of this problem in an early stage.

## Conclusion

From our experience we can conclude that conservative management is an effective method in the treatment of non-complicated acute mastoiditis, however myringotomy should be considered if there is no response within 48 hours, while cortical mastoidectomy should be used in conjunction with the medical management in the treatment of cases presented with subperiosteal abscess (with or without fistula), complicated cases, and in cases that showed failure of myringotomy.

## Consent

Written informed consent was obtained from the parents of the patients for publication of these cases and accompanying images. A copy of the written consent is available for review by the Editor-in-Chief of this journal.

## Competing interests

The authors declare that they have no competing interests.

## Authors' contributions

These authors contributed equally to this work and they read and approved the final manuscript

## Pre-publication history

The pre-publication history for this paper can be accessed here:

http://www.biomedcentral.com/1472-6815/10/1/prepub
